# The association between a body shape index and diabetic kidney disease in early-onset type 2 diabetes: evidence from a two-center study

**DOI:** 10.3389/fendo.2025.1553890

**Published:** 2025-06-19

**Authors:** Mengdie Chen, Yiyun Wang, Ping Feng, Lijing Wu, Hanying Liu, Yao Liang, Mengyao Yang, Qidong Zheng

**Affiliations:** ^1^ Department of Endocrinology, Taizhou Central Hospital (Taizhou University Hospital), Taizhou, China; ^2^ Department of Internal Medicine, Yuhuan Second People’s Hospital, Yuhuan, China

**Keywords:** a body shape index, visceral obesity, type 2 diabetes, diabetic kidney disease, early-onset

## Abstract

**Background:**

Early-onset type 2 diabetes mellitus (T2DM) is closely associated with an increased risk of diabetic kidney disease (DKD), and obesity is a well-recognized contributing factor. Traditional measures like body mass index (BMI) have limitations in capturing visceral fat distribution. The A Body Shape Index (ABSI), a newer anthropometric indicator, may provide a more accurate assessment of central obesity. This study investigated the relationship between ABSI and DKD in individuals with early-onset T2DM.

**Methods:**

This cross-sectional study analyzed data from 2,598 patients with early-onset T2DM enrolled at the National Metabolic Management Centers of Yuhuan Second People’s Hospital and Taizhou Central Hospital between 2017 and 2024. Multivariate logistic regression models were used to evaluate the association between ABSI and DKD, with additional analyses using restricted cubic splines (RCS) to explore dose-response patterns. Subgroup and interaction analyses were also performed.

**Results:**

Of the participants, 1,030 (39.6%) were diagnosed with DKD. The prevalence of DKD increased across ABSI tertiles: 35.8% in the lowest tertile (T1), 38.5% in the middle tertile (T2), and 44.7% in the highest tertile (T3) (P < 0.001). Higher ABSI was independently associated with a greater risk of DKD (OR = 1.24; 95% CI: 1.05–1.50; P = 0.022) after adjusting for potential confounders. Patients in the highest ABSI tertile had a significantly higher risk of DKD than those in the lowest tertile (OR = 1.24; 95% CI: 1.01–1.52; P = 0.041). RCS analysis showed a positive linear relationship between ABSI and DKD risk (P for non-linearity = 0.139), and the findings were consistent across subgroups.

**Conclusion:**

ABSI is positively and linearly associated with the risk of DKD in patients with early-onset T2DM. This metric may be useful for identifying individuals at higher risk and guiding early preventive strategies.

## 1 Introduction

Type 2 diabetes mellitus (T2DM) has traditionally been considered a condition affecting middle-aged and older adults (1). However, its incidence among younger populations has risen sharply in recent years (1). Early-onset T2DM, typically defined as a diagnosis before the age of 40, is associated with more severe clinical outcomes compared to late-onset T2DM (2). Individuals with early-onset T2DM face an elevated risk of cardiovascular disease (3), earlier mortality (4), and a faster progression of microvascular complications (5), including diabetic kidney disease (DKD) (6). The burden of DKD is particularly concerning in younger patients. Studies have shown that the cumulative incidence of end-stage kidney disease (ESKD) in T2DM is significantly higher in those diagnosed at younger ages, reaching 11.8% for diagnoses before age 30 and 9.3% for those diagnosed between ages 30 and 39 (6). These findings highlight the urgent need to understand better the risk factors contributing to DKD in early-onset T2DM and to improve risk stratification and prevention strategies in this vulnerable population.

Obesity is a well-established risk factor for DKD (7, 8), and body mass index (BMI) remains the most commonly used measure for assessing obesity (9). However, BMI has several limitations (10). It does not reflect fat distribution and cannot distinguish between fat and lean mass. To address these shortcomings, Krakauer and colleagues proposed the A Body Shape Index (ABSI), which incorporates waist circumference, height, and weight to better estimate central obesity (11). ABSI has been shown to capture visceral fat more accurately and has demonstrated associations with various metabolic disorders, including cardiovascular disease (12, 13), non-alcoholic fatty liver disease (14), metabolic syndrome (15), and diabetes (16). Recent studies have also reported significant associations between ABSI and kidney function impairment (17–19). However, data examining the relationship between ABSI and DKD in individuals with early-onset T2DM remains scarce.

To fill this gap, the present study analyzed data from 2,598 Chinese adults with early-onset T2DM to investigate the potential association between ABSI and the risk of developing DKD.

## 2 Materials and methods

### 2.1 Study design and participants

**Figure 1:**
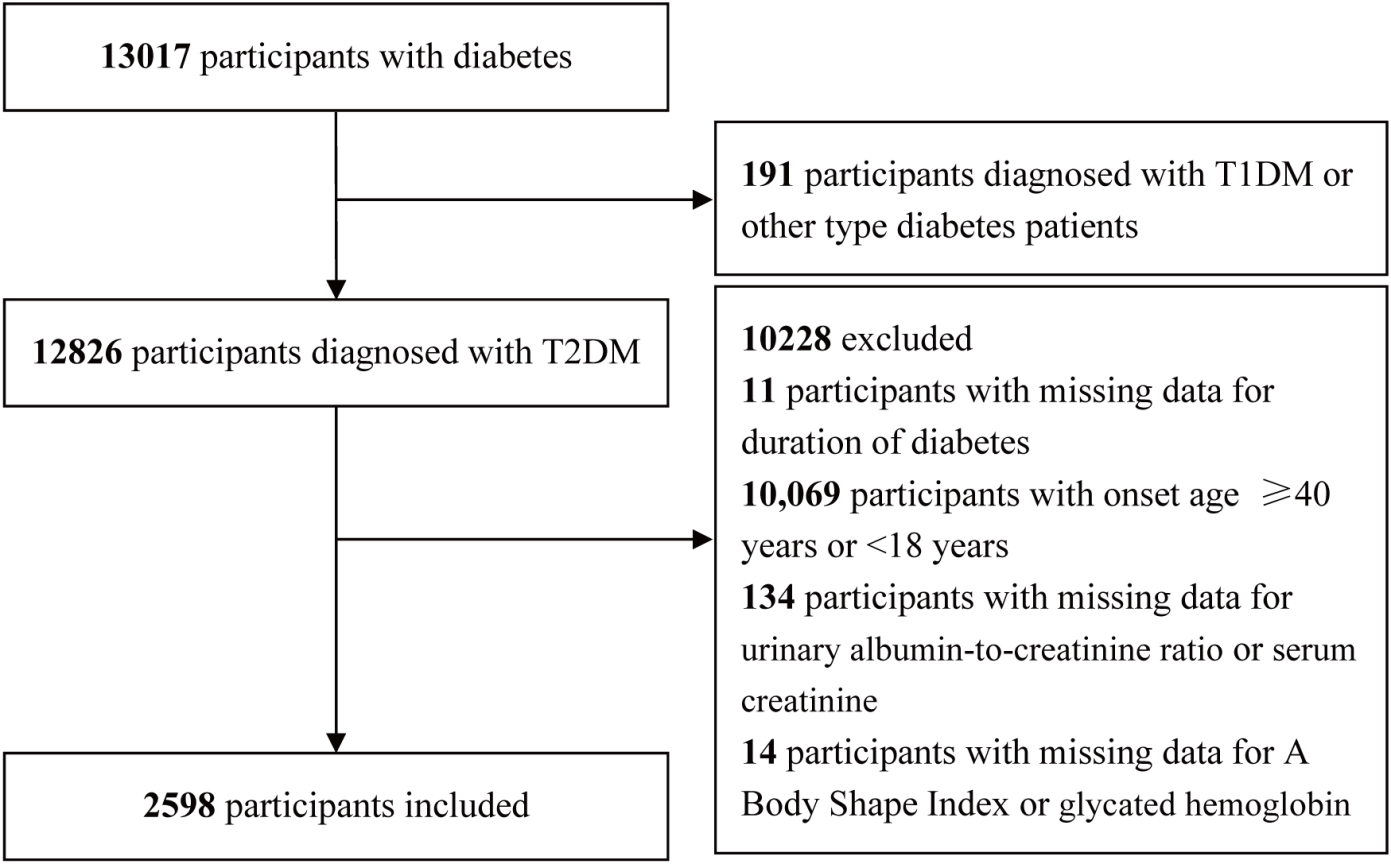
Study selection criteria.

This cross-sectional analysis made use of information from the National Metabolic Management Center (MMC) collected between September 2017 and May 2024. A comprehensive description of the MMC program can be found in previous publications (20–22). The Clinical Research Ethics Committees of Yuhuan Second People’s Hospital and Taizhou Central Hospital (Taizhou University Hospital) approved the study protocol, and written informed consent was obtained from all participants in a manner consistent with the Declaration of Helsinki. A comprehensive overview of the inclusion and exclusion criteria is presented in **Figure 1**. In total, 13,017 participants with diabetes were initially included. During the screening process, 191 participants diagnosed with other types of diabetes, such as type 1 diabetes mellitus (T1DM), 11 participants with missing data on diabetes duration, 10,069 participants with an onset age of diabetes ≥40 years or <18 years, 134 participants with missing data on the urinary albumin-to-creatinine ratio (UACR) or serum creatinine (Scr), 14 participants with missing data on anthropometric and biochemical parameters, including the ABSI or glycated hemoglobin (HbA1c), were excluded. A final cohort of 2,598 participants was retained.

### 2.2 Data collection and analysis

Patient data were collected with an independent digital medical record system detailed in our prior study (20–22). Collected data included age, sex, education level, smoking and drinking status, diabetes duration, family history of diabetes, hypertension, hyperlipidemia, coronary heart disease, stroke, blood pressure (diastolic and systolic), anthropometric measurements (weight, height, waist circumference), and laboratory values including fasting blood glucose (FBG), fasting serum C peptide (FCp), HbA1c, triglycerides (TG), total cholesterol (TC), low- and high-density lipoprotein cholesterol (LDL-c and HDL-c), urea nitrogen (UN), Scr, uric acid (UA), and UACR. Homeostasis model assessment of insulin resistance (HOMA-IR) calculations were performed with the following formula: 1.5 + (FCp [pmol/L] × FBG[mmol/L])/2800 (23). Estimated glomerular filtration rate (eGFR) calculations made use of the Chronic Kidney Disease Epidemiology Collaboration equation (24). BMI was measured as weight (kg) divided by height squared (m²). DKD was identified based on a UACR ≥30 mg/g and/or eGFR <60 mL/min per 1.73 m^2^ (25). The ABSI was calculated using the formula (11): WC(mm)*Weight(kg)^-2/3^*Height(m)^5/6^.

### 2.3 Statistical analyses

Continuous data are shown as mean ± standard deviation or median (minimum, maximum) when normally distributed and skewed, respectively. Categorical data are given as frequencies or percentages. These three respective data types were compared with t-tests, Mann-Whitney U tests, and chi-square tests. To evaluate the association between ABSI and diabetic kidney disease (DKD), logistic regression models were utilized to assess odds ratios (OR) with corresponding 95% (CIs). ABSI tertiles were used to establish three participant groups, with the first tertile serving as the reference group for all odds ratio calculations. Additionally, the trend in odds ratios across ABSI tertiles was assessed by treating ABSI as an ordinal variable. Four models were utilized to assess the relationship between ABSI and DKD: The crude model did not adjust for any variables, Model 1 involved adjustments for age and sex, while Model 2 included additional adjustments for diabetes duration, HbA1c, and smoking/drinking status, and Model 3 was additionally adjusted for hyperlipidemia, coronary heart disease, hypertension, and stroke. The nonlinearity of the association between ABSI and DKD was explored via restricted cubic spline (RCS) regression using knots at the 5th, 35th, 65th, and 95th ABSI percentiles. This analysis assessed both the linearity of the relationship and the dose-response pattern following adjustment for variables in Model 3. Interaction and stratified analyses were conducted according to sex (male or female), age (<40y or ≥40), education (below high school or high school and above), HbA1c (<7.5% or ≥7.5%), because levels above 7.5% indicating poor glycemic control in diabetic patients (26, 27), hypertension (yes or no), and hyperlipidemia (yes or no). Stratified logistic regression was utilized for subgroup analyses, with the likelihood ratio test being used to detect interactions among these subgroups. R 4.2.2 (http://www.R-project.org, The R Foundation) and Free Statistics software version 2.0 were used for all analyses, with a two-sided P < 0.05 being considered significant.

## 3 Results

### 3.1 Characteristics of participants

Overall, 2,598 participants were analyzed based on ABSI tertiles. Demographic, clinical, and metabolic factors differed significantly across the tertiles (**Table 1**). Males constituted the majority of the cohort (70.4%), with the proportion of males significantly different across tertiles (p < 0.001). Those in the highest tertile (T3) were significantly older relative to those in the first tertile (T1) (p < 0.001). In terms of anthropometric measures, WC increased across tertiles, while the opposite was observed for BMI (p < 0.001). Participants in the T3 group exhibited poorer glycemic control and higher insulin resistance, reflected by elevated FBG (p = 0.003), HbA1c (p = 0.037), and HOMA-IR (p = 0.002). UACR was also significantly greater in T3 relative to T1 (p < 0.001). DKD prevalence increased significantly from 35.8% in T1 to 44.7% in T3 (p < 0.001).

**Table 1:** Clinical features of participants.

Variables	ABSI				
	Total (n = 2598)	T1 (n = 866)	T2 (n = 866)	T3 (n = 866)	p
Sex, n (%)					< 0.001
Male	1828 (70.4)	565 (65.2)	664 (76.7)	599 (69.2)	
Female	770 (29.6)	301 (34.8)	202 (23.3)	267 (30.8)	
Age (y)	39.0 (34.0, 47.0)	37.0 (32.0, 43.0)	39.0 (34.0, 45.0)	42.0 (36.0, 52.0)	< 0.001
Onset age (y)	34.7 (30.8, 37.5)	33.7 (29.4, 37.0)	34.7 (30.8, 37.4)	35.5 (31.9, 37.9)	< 0.001
Education, n (%)					< 0.001
Below high school	1590 (61.2)	465 (53.7)	535 (61.8)	590 (68.1)	
High school education and above	1008 (38.8)	401 (46.3)	331 (38.2)	276 (31.9)	
DBP (mmHg)	77.0 (72.0, 86.0)	77.0 (72.0, 86.0)	77.0 (72.0, 86.0)	76.0 (71.0, 85.0)	0.008
SBP (mmHg)	127.0 (120.0, 138.0)	126.0 (119.0, 138.0)	127.0 (120.2, 138.0)	127.0 (120.0, 139.0)	0.281
BMI (kg/m2)	25.7 (23.1, 28.6)	26.0 (23.2, 28.9)	25.8 (23.6, 28.7)	25.1 (22.7, 28.1)	<0.001
WC (cm)	90.0 (83.0, 97.0)	85.7 (78.2, 92.0)	90.0 (84.5, 96.9)	93.0 (87.0, 100.0)	< 0.001
Duration of diabetes (y)	3.9 (0.1, 11.6)	2.2 (0.0, 9.5)	3.3 (0.1, 10.6)	7.1 (0.8, 16.1)	< 0.001
History of hypertension, n (%)	652 (25.1)	185 (21.4)	216 (24.9)	251 (29)	0.001
History of hyperlipidemia, n (%)	710 (27.3)	232 (26.8)	232 (26.8)	246 (28.4)	0.684
Coronary heart disease, n (%)	60 (2.3)	9 (1)	20 (2.3)	31 (3.6)	0.002
Stroke, n (%)	40 (1.5)	5 (0.6)	13 (1.5)	22 (2.5)	0.004
Family history of diabetes, n (%)	1466 (56.4)	493 (56.9)	484 (55.9)	489 (56.5)	0.909
Smoking, n (%)	959 (36.9)	285 (32.9)	363 (41.9)	311 (35.9)	< 0.001
Drinking, n (%)	1086 (41.8)	353 (40.8)	370 (42.7)	363 (41.9)	0.707
FBG (mmol/L)	9.3 (7.0, 12.8)	9.0 (6.8, 12.4)	9.6 (7.2, 13.1)	9.4 (7.1, 13.0)	0.003
FCp(ng/mL)	2.1 (1.3, 3.0)	2.0 (1.3, 3.0)	2.2 (1.4, 3.1)	2.0 (1.3, 2.9)	0.008
HOMA-IR	3.8 (2.9, 5.2)	3.7 (2.8, 5.0)	4.0 (3.0, 5.5)	3.8 (3.0, 5.0)	0.002
HbA1c (%)	9.2 (7.3, 11.2)	9.0 (7.1, 11.2)	9.3 (7.5, 11.2)	9.2 (7.5, 11.1)	0.037
UN (mmol/L)	4.8 (4.0, 5.9)	4.7 (3.9, 5.6)	4.9 (4.0, 5.9)	4.9 (4.1, 6.1)	< 0.001
Scr (mmol/L)	62.0 (51.0, 73.0)	62.0 (51.0, 73.0)	62.0 (52.0, 73.0)	62.0 (51.0, 73.0)	0.809
e-GFR (mL/min per 1.73 m2)	117.1 (97.7, 140.3)	116.6 (99.2, 138.8)	118.1 (101.1, 140.3)	115.6 (92.2, 141.8)	0.057
UA (mmol/L)	344.0 (278.0, 419.0)	339.5 (276.2, 417.0)	347.0 (279.0, 418.0)	348.0 (278.2, 421.0)	0.612
TG (mmol/L)	1.9 (1.2, 3.0)	1.7 (1.1, 2.8)	2.0 (1.3, 3.0)	1.9 (1.2, 3.1)	< 0.001
TC (mmol/L)	5.2 (4.4, 5.9)	5.1 (4.3, 5.8)	5.2 (4.4, 6.0)	5.2 (4.4, 6.0)	0.053
HDL-C (mmol/L)	1.0 (0.9, 1.2)	1.0 (0.9, 1.3)	1.0 (0.9, 1.2)	1.0 (0.9, 1.2)	0.021
LDL-C (mmol/L)	2.9 (2.3, 3.6)	2.9 (2.4, 3.6)	3.0 (2.3, 3.6)	2.9 (2.3, 3.6)	0.637
UACR(mg/g)	19.6 (8.2, 64.2)	15.9 (6.9, 56.0)	19.3 (8.7, 53.8)	23.9 (9.7, 79.3)	< 0.001
DKD, n (%)	1030 (39.6)	310 (35.8)	333 (38.5)	387 (44.7)	< 0.001
ABSI	0.080 (0.077, 0.082)	0.076 (0.074, 0.077)	0.080 (0.079, 0.081)	0.083 (0.082, 0.085)	< 0.001

Data are medians (IQR) or counts (%).

ABSI, a body shape index; T, tertile; SBP, systolic blood pressure; DBP, diastolic blood pressure; BMI, body mass index; WC, waist circumference; FBG, fasting blood glucose; FCp, fasting serum C peptide; HOMA-IR, homeostasis model assessment of insulin resistance; HbA1c, glycated hemoglobin; UN, urea nitrogen; Scr, serum creatinine; eGFR, estimated glomerular filtration rate; UA, uric acid; TG, triglycerides; TC, total cholesterol; HDL-C, high-density lipoprotein cholesterol; LDL-C, low-density lipoprotein cholesterol; UACR, urinary albumin-to-creatinine ratio; DKD, diabetic kidney disease.

### 3.2 Association between ABSI and DKD in early-onset T2DM

**Figure 2:**
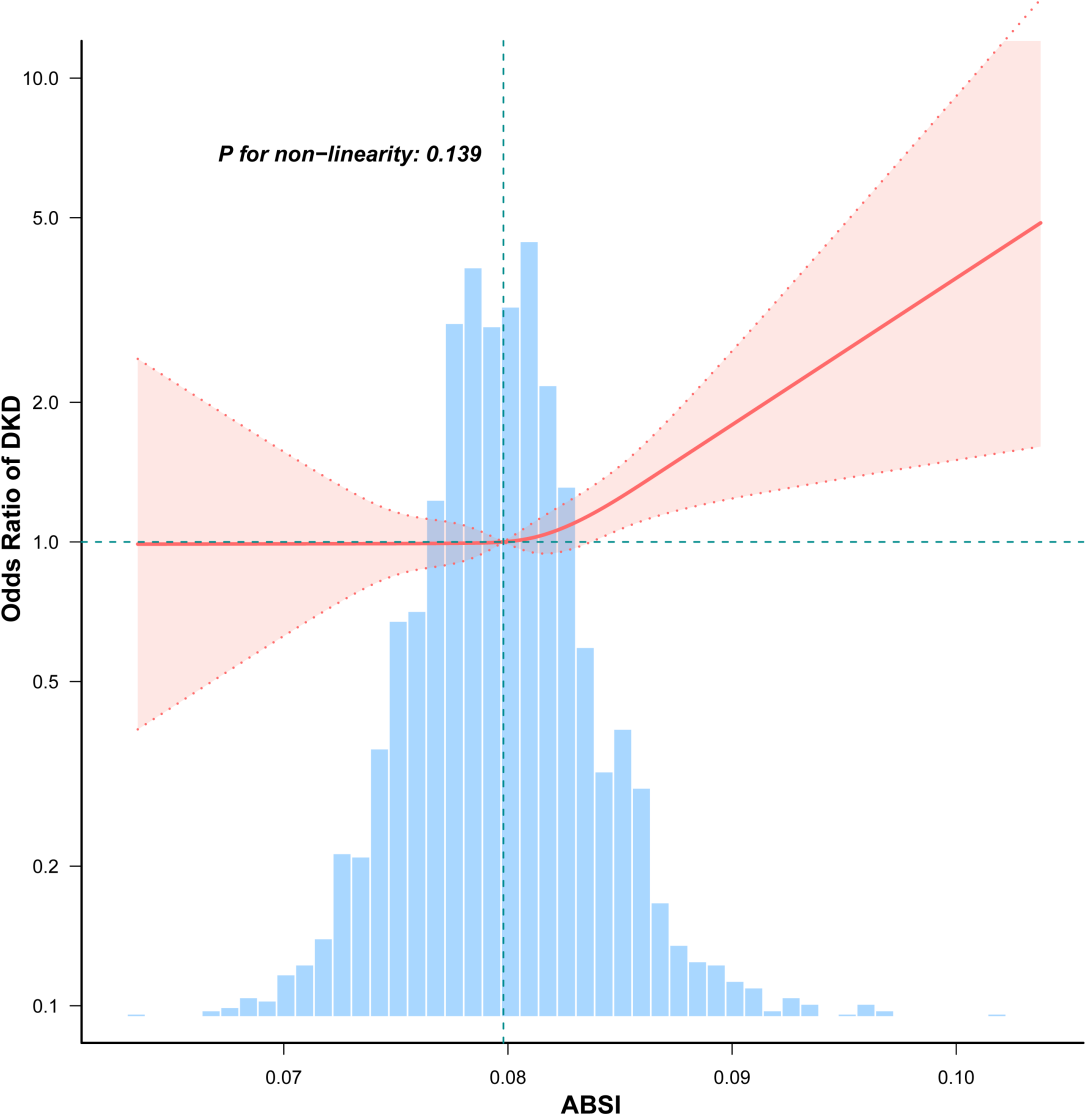
Non-linear association between ABSI and DKD in participants with early-onset T2DM. Adjusted for age, sex, duration of diabetes, HbA1c, drinking, smoking, hypertension, hyperlipidemia, coronary heart disease, and stroke, unless those were the variables used for stratification. The red solid line represents the fitted odds ratio estimated using restricted cubic spline regression. The shaded red area indicates the 95% CI. The histogram below illustrates the distribution of ABSI values among participants.

RCS analyses revealed that ABSI was linearly related to OR of DKD (P for non-linearity = 0.139). The odds of DKD increased significantly with higher ABSI values (**Figure 2**).

The association between ABSI and DKD was examined in a series of models adjusted for various factors (**Table 2**). When ABSI was analyzed as continuous variable, in the crude model, a 0.01-unit rise in ABSI was associated with a 46% rise in DKD risk(OR = 1.46, 95% CI: 1.22-1.75, P < 0.001). The significance of this relationship was retained after adjustments for basic demographic factors such as sex and age (Model 1: OR = 1.30, 95% CI: 1.08-1.55, P = 0.004). When adjusting for diabetes duration, HbA1c, smoking, and drinking (Model 2), the OR for DKD decreased slightly (OR = 1.26, 95% CI: 1.05-1.50, P = 0.011). When the model underwent full adjustment (Model 3) to account for comorbidities such as hypertension, hyperlipidemia, coronary heart disease, and stroke, this relationship remained significant (OR = 1.24, 95% CI: 1.03-1.48, P = 0.022). Further analysis by ABSI tertiles reinforced this finding. Cases in the top ABSI tertile (T3) had a 24% greater risk of DKD relative to cases in T1 under Model 3 (OR = 1.24, 95% CI: 1.01-1.52, P = 0.041). The trend across tertiles was significant (P for trend = 0.004).

**Table 2:** Association between ABSI and DKD in early-onset T2DM.

Variable	Crude	P value	Model 1	P value	Model 2	P value	Model 3	P value
ABSI (continuous)	1.46 (1.22-1.75)	<0.001	1.3 (1.08-1.55)	0.004	1.26 (1.05-1.5)	0.011	1.24 (1.03-1.48)	0.022
ABSI (categories)								
T1	1(Ref)		1(Ref)		1(Ref)		1(Ref)	
T2	1.12 (0.92-1.36)	0.253	1.05 (0.86-1.28)	0.654	1.04 (0.85-1.27)	0.721	1.02 (0.83-1.25)	0.831
T3	1.45 (1.19-1.76)	<0.001	1.27 (1.04-1.55)	0.018	1.25 (1.02-1.52)	0.032	1.24 (1.01-1.52)	0.041
P for trend		<0.001		0.017		0.032		0.04

Continuous variable: ABSI was scaled by a factor of 100, with the OR corresponding to the change in DKD risk for every 0.01 rise in ABSI. Crude: unadjusted; Model 1: adjusted for sex, age; Model 2: adjusted for Model 2 + duration of diabetes, HbA1c, smoking, drinking; Model 3: adjusted for Model 3 + hypertension, hyperlipidemia, coronary heart disease, stroke.

**Figure 3:**
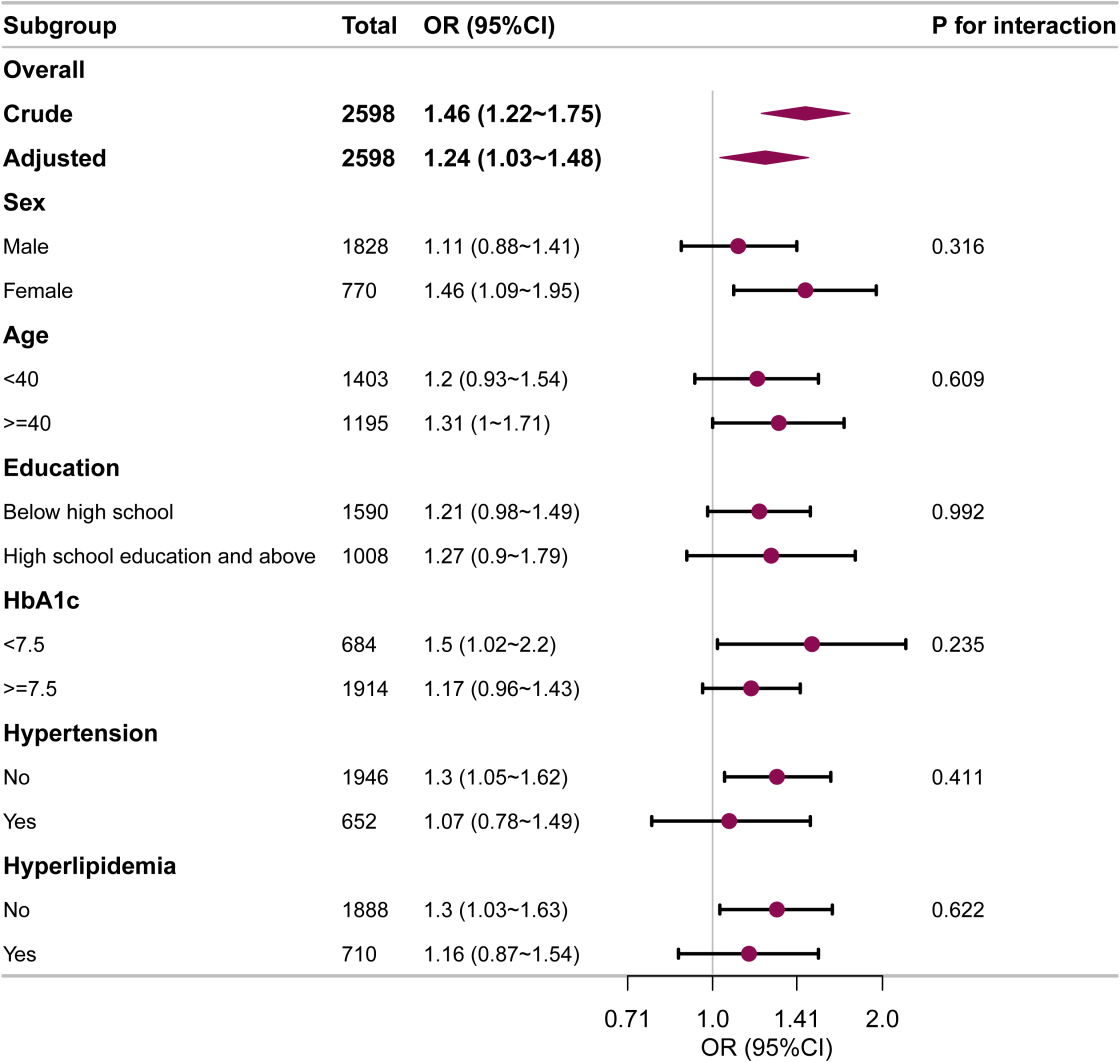
Subgroup and interaction analyses for the relationship between ABSI and DKD in early-onset T2DM. Adjustments were made for age, sex, diabetes duration, HbA1c, drinking, smoking, hypertension, hyperlipidemia, coronary heart disease, and stroke, unless those were the variables used for stratification. OR, odds ratio; CI, confidence interval.

Subgroup analyses were also undertaken to evaluate the relationship between ABSI and DKD in various subgroups, including sex, age, education, HbA1c, hypertension, and hyperlipidemia (**Figure 3**). The analyses revealed that higher ABSI values were consistently linked with elevated DKD risk across all subgroups. No significant interactions were found in any subgroups.

## 4 Discussion

In this study, we conducted a multivariable logistic regression analysis to assess the association between ABSI values and the risk of DKD in patients with early-onset T2DM, while controlling for potential confounding factors. The results of the logistic regression indicated that higher ABSI values were significantly associated with an increased risk of DKD. RCS curves revealed that ABSI and DKD were positively associated with one another in a linear relationship, and these findings remained robust in subgroup analyses.

Compared to BMI, ABSI is considered a more accurate predictor of health risks associated with abdominal obesity because it more effectively captures the distribution pattern of visceral fat. In recent years, research has found a significant relationship between ABSI and kidney disease. Kim et al. (28) showed that ABSI was more strongly related to CKD than BMI was. ABSI is better at distinguishing different stages of CKD than BMI. ABSI also has a higher predictive ability for moderate to severe CKD compared to BMI. Sun et al. (17) determined that ABSI was positively correlated with UACR. Su et al. (29) determined that high ABSI was related to rapid renal functional decline. Zhang et al. (19) found that following adjustment for confounding factors, ABSI quartiles were positively related to elevated UACR (OR [95% CI] Q4 vs. Q1: 1.183 [1.080, 1.295], p for trend < 0.001). Our study focuses on the relationship between ABSI and DKD, and a positive correlation was also identified. A Mendelian randomization study (18) showed that a rise of one standard deviation in the value of genetically predicted ABSI led to increased UACR in women (β = 0.039 [95% CI: 0.016, 0.063] log [UACR], P = 0.001 for ABSI), but not in men. Yu et al. (30) also found that in women, ABSI values in those with CKD were markedly elevated relative to non-CKD cases, whereas the same was not true for men. Stratified analysis further revealed that as ABSI increased, elevated UACR was more likely to occur in populations characterized by older age, male gender, and hypertension. However, the study by Cosoreanu et al. (31) found that among White patients, ABSI was related to an extremely high risk of CKD progression, while no such association was found in Romani patients. The subgroup analyses in this study demonstrated stable results, with no significant interaction effects detected across the subgroups.

CKD is more common and progresses faster in obese diabetic patients compared to those with normal weight (32). This is a primary factor explaining the higher cumulative CKD incidence in T2DM patients as compared to those with T1DM (33). Obesity has a negative effect on risk factors for CKD, such as glycemic control, hypertension, and lipid levels, contributing to insulin resistance. It can also directly impact the kidneys, altering glomerular hemodynamics, increasing sympathetic nervous activity, and contributing to altered growth factor expression, systemic inflammatory activity, endothelial dysfunction, hypertension, and visceral fat-related kidney compression (34). Even without comorbid diabetes, obesity has been linked to greater proteinuria frequency and severity, with many reports of obesity-related glomerular lesions to date (35).

Our study revealed that ABSI and DKD are positively related to one another in patients with early-onset T2DM, suggesting that ABSI may be an important biomarker for screening the risk of developing DKD. Our study included a large participant population, and our data analysis strategy was comprehensive and methodologically rigorous. We employed multivariable regression with adjustment for potential confounding factors, ensuring that the observed link between ABSI and DKD was not influenced by other variables. Additionally, the use of generalized additive models and smooth curve fitting enabled explorations of potential nonlinear relationships between ABSI and DKD, providing a more nuanced understanding of this association. The robustness of the subgroup analysis further supports the stability of our results. Overall, the combination of a large, well-defined cohort and complex statistical techniques strengthens the credibility of our study and its potential implications for clinical practice. Given the increasing prevalence of T2DM and DKD in younger populations, our findings provide unique insights into the relationship between ABSI and DKD, filling a gap in the current literature, as there is limited research on anthropometric indicators as predictors of early-onset T2DM-related DKD. Future studies can further explore the mechanisms behind the ABSI-DKD relationship, particularly through longitudinal studies, to better understand its predictive value and how it can be integrated into broader public health strategies to reduce the burden of diabetes-related complications.

This study has limitations. First, the study involved only two centers, potentially limiting the generalizability of the findings. Therefore, future studies should be conducted in a broader multi-center setting to validate the generalizability and applicability of our findings. Second, as an observational study, while we made efforts to control for measurable confounding variables in the data analysis, the potential impact of unmeasured or unrecorded confounders cannot be excluded. For instance, in the absence of genetic testing, we cannot entirely rule out the possibility of maturity-onset diabetes of the young (MODY) in the participants. Third, the study analyzed a Chinese population, and extending the findings to any other ethnicities warrants caution. Differences in metabolism, genetics, and lifestyle factors across populations could potentially affect the relationship between ABSI and DKD. Finally, since this study is cross-sectional, the results should be interpreted cautiously as causal inferences cannot be drawn. Additional prospective studies are crucial to evaluate the predictive ability of ABSI for DKD risk.

## 5 Conclusions

In summary, ABSI and DKD were herein found to be significantly and positively linearly related to one another early-onset type 2 diabetes. ABSI may offer utility for establishing which individuals face a high risk of DKD, enabling earlier interventions. However, further prospective studies will be necessary to confirm causality and explore the long-term clinical impact.

## Data Availability

The raw data supporting the conclusions of this article will be made available by the authors, without undue reservation.
